# Emerging Pests and Vector-borne Diseases in Europe

**DOI:** 10.3201/eid1411.080945

**Published:** 2008-11

**Authors:** Alfons Van Gompel, Wim Van Bortel

**Affiliations:** Institute of Tropical Medicine, Antwerp, Belgium

**Keywords:** Vector-borne parasites, ectoparasites, bluetongue, chikungunya, book review

Today’s hot topic is the risk of introducing new vector-borne diseases and harmful ectoparasites into Europe, or of the geographic extension of existing ones. The recent outbreaks of bluetongue virus infection in northwestern Europe and of chikungunya infection in Italy are real reminders of the vulnerability of this ill-prepared continent, where the idea prevails that these things cannot happen there but are merely problems that occur on other continents.

This book is the first volume of a new series, “Ecology and Control of Vector-Borne Diseases,” written by more than 70 European scientists who are recognized experts in their specific fields. The cover text states that in 24 chapters “this book provides examples of the most likely pests and diseases affecting man and animals in Europe, with emphasis on ecological factors favoring these diseases and methods for prevention and intervention.” Indeed, the raised expectations are largely fulfilled.

The book is divided into 6 sections preceded by an appealing introductory chapter—Alarm Bells Ringing: More of the Same, and New and Novel Diseases and Pests—and followed by a poignant epilogue, which summarizes the different problem areas discussed in the book as well as concise actions proposed to reduce the threats.

In the first section, pertinent questions are answered on the possible return of malaria in Europe and on the problem of leishmaniasis in southern Europe. Avian malaria, a rather unknown but interesting paradigm, is discussed as well. In the second section, different arboviruses such as bluetongue, West Nile, chikungunya, and dengue are discussed in addition to the rather unknown Usutu virus. Readers can download the well-written chapter on chikungunya and dengue in southern Europe from www.wageningenacademic.com. The third section gives an overview on the current problems of tick-borne encephalitis and Lyme disease in Europe; discussion is limited to the Baltic States and the Netherlands, respectively. The fourth section discusses strategies on the following emerging arthropod pests and problems: psoroptic mang, the establishment and spread of the *Aedes albopictus* mosquito in Europe, bed bugs, houseflies, head lice, and *Culicoides* midges. The authors highlight the most recent information on pest control with an emphasis on tools other than classic chemical control to manage these infestations because these pests are likely to escape these classic chemical tools in the future. In the fifth section, Surveillance, Protection and Control, the authors discuss monitoring systems for adult insect pests and disease vectors, personal protection against European disease vectors, and mosquito control in Europe. The last section, Nature Conservation, Wildlife Management and Human Activities as Drivers, covers subjects such as changes in global scale land use and its implications for nature conservation and emerging vector-borne diseases, wildlife and the risk of vector-borne viral diseases, and invasions of vector-borne diseases driven by transportation and climate change.

Throughout the book, the role of the different factors that drive the changing epidemiology of diseases and pests is described in a well-balanced manner. This changing epidemiology is recognized as a possible consequence of the complex interplay of factors such as climate change (whether or not it is anthropogenic), human-made environmental change (e.g., agricultural activities), increasing international trade and traffic (e.g., long-distance tourism), changes in human behavior (e.g., more outdoor activities), and the development of insecticide resistance.

It was a rewarding task, as well as a pleasant challenge for us, a physician and a biologist/entomologist, to read the book from cover to cover. The volume is not intended to be read as a novel, but every chapter is certainly well worth reading. The book is written for the nonexperienced (as an introductory text) and for the experienced scientist (as a refresher regarding knowledge in a specific domain). Because it contains chapters on human and animal diseases and on pests, it can help broaden the horizon for every concerned scientist who might not be accustomed to the problems outside of a particular discipline.

The information on the different topics has increased since the authors finished writing it (end of September 2007), but this outstanding book is a useful beginning for the novice who wants to acquire, in a relatively short period, a nearly complete insight on the existing information concerning pests and vector-borne diseases in Europe. Although this book does not cover everything (e.g., rickettsial diseases were only superficially discussed), it nevertheless deserves a place on the bookshelf of every concerned infectious diseases specialist or epidemiologist, scientist or student.

